# Editorial: Reviews in behavioral and lifestyle interventions for healthy aging: 2022

**DOI:** 10.3389/fpubh.2024.1354881

**Published:** 2024-02-05

**Authors:** Hiroto Narimatsu, Lei Feng

**Affiliations:** ^1^Graduate School of Health Innovation, Kanagawa University of Human Services, Kawasaki, Japan; ^2^Cancer Prevention and Control Division, Kanagawa Cancer Center Research Institute, Yokohama, Japan; ^3^Center for Innovation Policy, Kanagawa University of Human Services, Kawasaki, Japan; ^4^Department of Genetic Medicine, Kanagawa Cancer Center, Yokohama, Japan; ^5^Department of Psychological Medicine, Yong Loo Lin School of Medicine, National University of Singapore, Singapore, Singapore; ^6^Centre for Healthy Longevity, National University Health System, @AgeSingapore, Singapore, Singapore; ^7^Healthy Longevity Translational Research Program, Yong Loo Lin School of Medicine, National University of Singapore, Singapore, Singapore

**Keywords:** Information and Communication Technologies (ICT), healthy aging, non-communicable chronic diseases, preventive medicine, health longevity

Non-communicable diseases (NCDs) are a primary global healthcare concern. They are often caused by a combination of genetic, physiological, environmental, and behavioral factors (1). Preventing NCDs can reduce the risk of intrinsic capacity decline. Thus, the World Health Organization (WHO) has introduced a “Healthy Aging” policy that focuses on maintaining an individual's functional ability by combining their intrinsic capacity with environmental characteristics (2). An individual's health status is determined by multiple factors, such as lifestyle, mental health, and community participation. Therefore, quantifying a person's health status and identifying modifiable factors to prevent age-related conditions are essential to effectively promote healthy aging. Maintaining motivation to sustain healthy activities and lifestyles is a crucial concern. Conventional healthcare interventions can often be tedious or unenjoyable, making it challenging to adhere to them long-term. Consequently, innovative and optimal strategies must be implemented to ensure continued success. Our Research Topic contains exciting articles on behavioral and lifestyle interventions that can contribute to healthy aging.

A Kanagawa University of Human Services research group (Nakamura et al.) conducted the ME-BYO index project. The ME-BYO index can comprehensively and numerically measure and visualize an individual's health status and future disease risk by quantifying data on the four domains of metabolic function, locomotor function, cognitive function, and mental resilience. This measurement could be optimal for motivating people to lead a healthy lifestyle. Considering psychological disorders are a major concern in healthy aging, the ME-BYO index includes mental resilience. Xu et al. focused on horticultural therapy for older adults with depression and demonstrated its effectiveness through a meta-analysis. This approach may be another option for healthy aging. Promoting the social participation of older adults is a promising approach to improving adult's ME-BYO status. Volunteer programs, reported by Zhu et al. are recognized as effective, even for older adults with cognitive impairments. Interestingly, remote programs can also be a helpful alternative. The coronavirus (COVID-19) pandemic highlighted the potential of Internet technology in health promotion. However, further studies using these techniques are required to test its effectiveness.

Furthermore, pets may play a vital role in healthy aging. A scoping review by Taeckens et al. demonstrated the effects of pet ownership on health. This traditional approach has considerable room for improvement for application in healthy aging. The authors concluded that more research is needed to determine how human-animal interactions may promote health among older adults. This information can help implement various effective approaches involving animals. Another issue is the motivation to maintain self-health. To overcome this, mindfulness could be an alternative and attractive approach. Li et al. conducted a meta-analysis and found consistent support for mindfulness practices related to promotion of motivation for self-health, especially intrinsic motivation. However, this review included studies using heterogeneous methods, indicating the need for future studies to arrive at definitive conclusions (Li et al.).

Behavioral and lifestyle interventions for healthy aging must be conducted from diverse perspectives. Numerous trials and errors are essential for establishing optimal approaches. This Research Topic included unique and promising approaches that will promote effective and sustainable interventions for preventing non-communicable diseases, contributing to research on healthy aging.

## Author contributions

HN: Conceptualization, Writing—original draft, Writing—review & editing. LF: Conceptualization, Writing—review & editing.
